# An agent-based algorithm resembles behaviour of tree-dwelling bats under fission–fusion dynamics

**DOI:** 10.1038/s41598-020-72999-0

**Published:** 2020-10-08

**Authors:** Ján Zelenka, Tomáš Kasanický, Ivana Budinská, Peter Kaňuch

**Affiliations:** 1 Institute of Informatics, Slovak Academy of Sciences, 845 07 Bratislava, Slovakia; 2 Institute of Forest Ecology, Slovak Academy of Sciences, 960 53 Zvolen, Slovakia

**Keywords:** Ecological modelling, Animal behaviour, Computer science

## Abstract

Utilization of computational approach in the study of social behaviour of animals is increasing and we attempted such an approach in our study of tree-dwelling bats. These bats live in highly dynamic fission–fusion societies that share multiple roosts in a common home range. The key behavioural component associated with complex and non-centralized decision-making processes in roost switching is swarming around potential locations in order to recruit members to the new roost. To understand roost switching dynamics of bat groups in their natural environment, we employed a computational model, the SkyBat, which is based on swarm algorithm, to model this process. In a simulated environment of this agent-based model, we replicated natural fission–fusion dynamics of the Leisler’s bat, *Nyctalus leisleri*, groups according to predefined species and habitat parameters. Spatiotemporal patterns of swarming activity of agents were similar to bats. The number of simulated groups formed prior to sunrise, the mean number of individuals in groups and the roost height did not differ significantly from data on a local population of bats collected in the field. Thus, the swarm algorithm gave a basic framework of roost-switching, suggesting possible applications in the study of bat behaviour in rapidly changing environments as well as in the field of computer science.

## Introduction

Group living has numerous energetic and social benefits for animals^1,2^. The survival rate of bats primarily increases through communal breeding and offspring rearing^3^. Bats living in social groups depend strongly on the protective function and microclimatic suitability of daily roosts^4^, with a significant proportion of more than 1400 species worldwide roosting in cavities of trees^5–7^. Maternity groups of most of tree-dwelling bat species change their roosts every couple of days, and the distance between two such roosts may exceed hundreds of metres in a dense forest environment^8–14^. While bats have perfect homing abilities and complex spatial orientation^15^, group membership in tree-dwelling species often alternates after evening emergence^3^. As a result, these bats live in highly dynamic fission–fusion societies in which they split up and rejoin larger or smaller groups of conspecifics, sharing multiple roosts in the common home range^16–21^.

Tree hollows excavated by woodpeckers or even more ephemeral shelters beneath loose bark provide limited room for maternity groups of tree-dwelling bats, especially considering their rapid doubling in size after the births of pups in midseason^5^. Therefore, frequent shuffling of adult females and their offspring among groups maintains long-term social relationships within their colony^3,22^. Tree roost characteristics are in relationship with ambient conditions^23^, thus roost switching ensures also microclimate that is most conducive to successful reproduction^24–26^. This behaviour also reduces parasites in the cavities, minimizes commutes and competition with other cavity dwellers and protects against predators that can deplete bat colonies if shelters are occupied for a longer time^18,26–28^.

The key behavioural component associated with roost switching and roost-mate recruitment in tree-dwelling bats is a swarming behaviour in which individuals eavesdrop on calls of conspecifics at multiple roosts within the colony home range (In order to make terminology clear, the more common use of the term swarming in bat biology is associated with pre-hibernation mating behaviour. In our case we refer to so-called ‘dawn swarming’^29^). Colony members perform a set of characteristic flight displays accompanied by social vocalisation and calls with long-distance propagation attributes in front of potential roosting sites, beginning at night and reaching its peak at dawn^30–37^. This unique behavioural mechanism is believed to drive the collective selection of new roosts from a large set of potential roosting sites. A simplified process of roost switching includes disintegration of the former group during foraging along with an individual search for a roost, swarming around potential locations in order to recruit other members and to select a new roost once the number of gathered mates is acceptable. Thus, roost switching is a multi-factorial task in which each bat evaluates the size of their group, quality of the roost, and remaining time to feed^5,36,38^.

This decision-making process observed in roost switching in tree-dwelling bats suggests a computational algorithm, a set of rules that defines a sequence of instructions to rule cooperation in a multi-agent system. Modern computational approaches to the study of social behaviour of animals are increasingly used and this emerging field provides many similar agent-based models today^39–42^ even in social behaviour of bats^43–46^. The first agent-based model that attempted to simulate roost switching in tree-dwelling bats using biologically relevant data was run by Ruczyński and Bartoń^38^. The model based on predefined assumptions has found that the bats that were successful in finding roosts were mainly those possessing accurate tree discrimination, i.e. the ability to quickly and effectively assess trees for suitability, whereas bats without this ability instead used the tactic of eavesdropping on swarming conspecifics in large groups. Another data-driven modeling of group formation in the fission–fusion dynamics of bats worked on a system of roosts of fixed characteristics with accounted pair-specific social influence^47^. However, it is questioning how collective decision is made under variable roost conditions and unstable social relationships. Deficiencies of previous models may be solved with the use of the swarm algorithm SkyBat, inspired by fission–fusion societies of bats^48,49^. Among the unique features of this computational algorithm is the ability to perform group movement without a need of individually specific exploration behaviour and social influence while searching for new targets of interest and hence an ability to perform flexible, non-centralized group decision-making in rapidly changing environments. Thus, the SkyBat model does not emphasise individual tree discrimination ability because tree roosts are ephemeral, and suitability of a cavity may change quickly during the course of season^14,18,21,50^. Moreover, this model assumes that each bat regardless of its relationships with other members must find a group in a suitable roost within a certain time limit for activity, which enables the algorithm to comprehend and simulate roost switching dynamics of bat groups in their natural environment.

In this study, we aimed to test the robustness of the SkyBat algorithm, specifically its ability to replicate natural fission–fusion dynamics of bat groups in specific conditions. We adjusted the first version of this model^48^ and fed the algorithm according to the set of basic rules and properties of bat movement, group sizes, roosting preferences and swarming behaviour observed in the field. This led us to simulate roost switching behaviour of tree-dwelling Leisler’s bats, *Nyctalus leisleri*, in a defined time frame and environment akin to real roosting areas.

## Material and methods

### Field data collection

**Figure 1:**
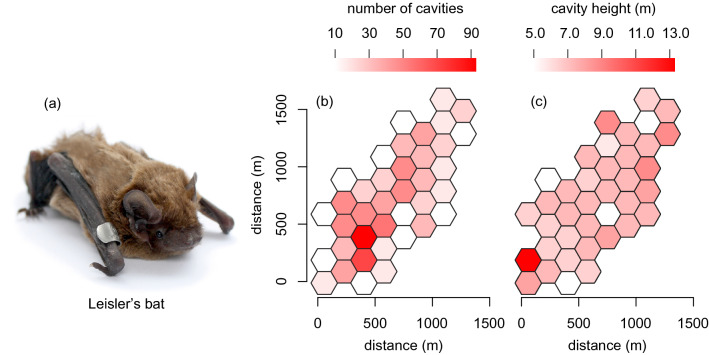
(**a**) A female of tree-dwelling Leisler’s bat, *Nyctalus leisleri*, from the study population. Photo credit: Peter Kaňuch. (**b**) The number of tree cavities and (**c**) the mean elevation of cavity entrance above the ground in the old pastured oak woodland. Population size was estimated to ~ 100 adult females in this area.

In order to obtain natural data that could be compared with our computer simulations, we studied a population of the Leisler’s bat, *Nyctalus leisleri*, breeding in old pastured oak woodland in Gavurky Protected Area (Pliešovská Kotlina Basin, central Slovakia; N48° 27′ 50.906″, E019° 7′ 48.534″; 460 m a.s.l.). This species is a medium-sized (body mass 13–18 g), insectivorous, migratory vespertilionid bat (Fig. 1a) inhabiting a predominantly forested landscape in a temperate zone throughout most of Europe^50^. Their breeding season is between May and August, during which maternity groups of adult females and their young switch their tree roosts every few days^11,36^. Groups roosting in tree cavities in Gavurky were found by radio-tracking or by search during dawn swarming with the help of a bat detector, and they were censused at evening emergence, supplemented by harp-trapping and individual banding. During long-term field observation from 2003 to 2014, we collected data on spatial distribution of bats’ roosts, group size, elevation of cavity entrance above the ground (hereafter cavity or roost height) and distance travelled during group roost switching (for details see^35,36,51–54^). All methods were carried out in accordance with relevant guidelines and regulations. All experimental protocols were approved by the Ministry of Environment of the Slovak Republic.

### Agent-based model settings

#### Bats

According to our field observations, a conservative estimation of the local population size was one hundred individual bats. Using this data, we designed a comparable number of agents in the model to replicate generic individuals of the Leisler’s bat group, including characteristics regarding species-specific flying, foraging and behaviour. This model only simulates adult females in groups without juveniles (which are typically delivered midseason), though newly volant offspring do roost with their mothers^16,19,20^.

Agents moved within the simulated environment by highly correlated random walk, following nearly linear path. The direction and flight speed of an agent at each step was randomly chosen within predetermined limits. To maintain biological relevance, the values for flight speed were derived using roughly Poisson distribution with a minimum flight speed of 2.5 m s^−1^ (the take-off velocity in similar sized species^55^), a median of 3.7 m s^−1^ and a maximum of 11.1 m s^−1^^56^. Movement of bats was described by the following equation:

1 $${\overline{v}}_{m}\left(t\right)={\overline{v}}_{d}\left(t\right)+{\overline{v}}_{s}\left(t\right)$$

where *v*_*m*_ is a bat movement vector, *v*_*d*_ is the distance travelled by random speed and *v*_*s*_ is random vector of the swerve (Fig. S1 of Supplementary Information). To detect a cavity, we set the field of perception (*FoP*) of bat agents at an angle of 270° and a distance of 150 m in accordance with structural characteristics of echolocation calls of the species^50^. To avoid the counter effect of atmospheric attenuation, bats usually tune to lower frequencies that are more resistant but energetically very costly^57^. We estimated that the low frequency calls of Leisler’s bats during swarming are audible to other eavesdropping individuals at a distance of 80–100 m (*D*_*attr*_). Swarming agents that were attracting roost-mates made such calls in a variable radius *D*_*attr*_ around the cavity in order to lure in any individuals randomly passing this circle.

#### Environment

For roost switching simulations, we designed an area of 128.2 ha (~ 900 × 1800 m) with a 3-dimensional configuration identical to habitat conditions in the field (*x*, *y* coordinates and *z* heights of 944 cavities available for roosts). This simplified habitat setting was considered sufficient because cavity density and height are the primary determining factors in roost-site selection of tree-dwelling bats^6,10,11,25,54^. In a hexagonal grid divided into 37 equal cells, density of cavities ranged from 8 to 92 per a cell (Fig. 1b) and mean cavity height from 4.8 to 13.5 m (Fig. 1c). This roosting habitat was centralized in a square plane (9000 × 9000 m). The total dimensions of our simulation environment were set with respect to the possible distance the species might travel while foraging^50^.

In general, this species prefers higher tree roosts^10,11,25,54^. On the other hand, it should avoid roosts infested by parasites^18,26^; therefore, excrements were used as a proxy of the load of parasites associated with guano^28^. The quality of a roost (*QR*), thus the attractiveness of a tree cavity for roosting, was expressed by the equation:

2 $${QR}_{i}\left(t\right)=\left(\left(\frac{100*{CH}_{i}}{\underset{i}{max}\left(CH\right)}\right)*{w}_{CH}\right)+\left(\left(-1*\left(LE\left(t\right)-100\right)\right)*{w}_{LE}\right)$$

where *CH* was cavity height, *LE* was the load of excrements (0–100) and *w* was the weight of the cavity height or load of excrements. In this simulation, both weights were set by default to 0.5. With each second of simulation, the value of *LE* increased by 5 × 10^–5^ times the number of bats in a roost during the course of a day. Effect of excrements decreased by a value of 10 if the cavity was empty (until zero) and increased by 0.1 times the number of bats in occupied roosts (until 100).

#### Temporal settings

The length of a season was set to 120 days, which should cover the full breeding period of the species in the study area. This period begins in early May when adult females arrive from hibernacula in southern Europe, and it ends in late August when offspring leave the maternity colony as the colony breaks apart. For the sake of simplicity of computation, we did not consider seasonal variation in the daytime. The length of time available to study the species with nocturnal and crepuscular activity outside the roost was fixed to eight hours per day (one cycle), which is an average nighttime during the summer at this latitude. One simulation step in the cycle was equal to 1 s, with 28,800 steps per cycle in total. In the beginning of the simulation, the whole group was located in one randomly chosen roost. After evening emergence from this roost, a foraging period began, which might vary from two to three hours per individual. During this period, bats were foraging within the total area of the environment, neither interacting with the environment nor with each other. This ensured that all agents were haphazardly distributed within the environment before the swarming period, which can take most of the night^36^.

#### Model interactions

Model agents implemented a non-centralized group decision process in which the group searched for a new roost based on their quality and attracted other roost-mates to form a suitable group. Bats often leave roosts when directly attacked by predators^26^, thus at the same time, groups worked to eliminate predation risks that was expected higher if roost switching is over a short distance^10,27^.

At the end of each foraging period, agents selected one roost from the set of cavities located within their *FoP*. Once the *QR* was satisfactory (*QR* > 30), the individual would begin signalling to attract potential group members by swarming around the cavity. A roost with at least one signaller already swarming around it had a higher probability that another agent, a receiver or non-signaller, would select it, if this occurred within the *D*_*attr*_ buffer. Receivers locating multiple roosts within the *D*_*attr*_ area would select the roost with the largest number of signallers. The length of time (*t*_*attr*_) the signaller spent at a roost in order to attract other agents was calculated as follows:

3 $${t}_{attr}\left(t\right)=\left({CH}_{i}^{2}+1\right)+NRB\left(t\right)+\frac{1000}{TTS}$$

where *CH* was cavity height, *NRB* was the number of bats in the roost and *TTS* was time to sunrise. The length of additional time (*te*_*attr*_) for signalling primarily depended on the number of other co-swarming bats and was calculated as follows:

4 $${te}_{attr}\left(t\right)=\frac{{NSB}^{2}\left(t\right)}{2}$$

where *NSB* was the number of signalling bats swarming at the roost. After this time, the signaller would enter the roost and remained inside as long as another signaller was still attracting new roost-mates or, if the group had already reached threshold size (*GST*), until the next evening emergence. This was expressed by the equation:

5 $$GST\left(t\right)=\left\{\begin{array}{ll}b& t\in \langle 0,o\rangle \\ b-\frac{\left(b-a\right)*(t-o)}{{T}_{sim}-t}& t\in \langle o,{T}_{sim}\rangle \end{array}\right.$$

where the *a* and *b* parameters represented the minimum and maximum values of group size (10 and 100 individuals, respectively, in the simulated population), *T*_*sim*_ was the time of simulation cycle and *o* was the time (set to midnight) when the signalling agents’ efforts into increasing the group size began decreasing. Once the signalling time reached defined limits and the group size did not reach required *GST*, all bats left their cavity and began searching for other roosts either as signallers or receivers.

**Figure 2:**
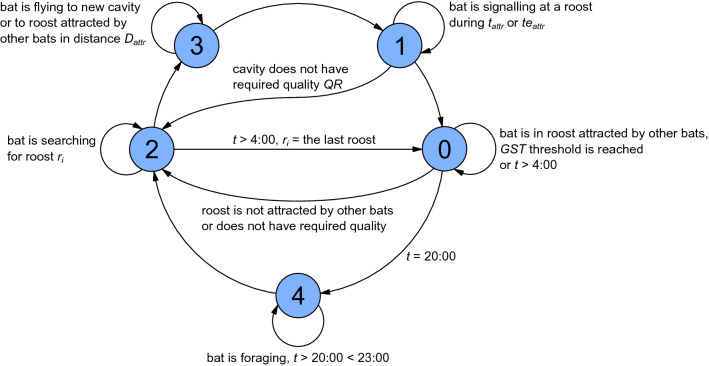
State diagram of an agent-based behavioural model designed for finding a suitable roost for a bat group under fission–fusion dynamics (0 – bat is in the roost; 1 – bat is signalling the roost to other bats; 2 – bat is searching for roost; 3 – bat is flying to new cavity or to attracted roost; 4 – bat is foraging).

Our model also acknowledged the role of bats’ memory in the decision-making process. Signalling the same roost or nearby roosts repeatedly over multiple nights was thus penalized because of the higher risk of predation^10,27^. This penalization was implemented on roosts within a 0 to 150 m radius around the roost. The distance (*PD*) that penalized repeated selection of the actual location increased with time, while *PD* for previously used roosts decreased (Fig. S2 of Supplementary Information). Thus, inspection of a cavity that did not already have a signaller (control of *QR*) depended on a number of hypotheses. In general, these hypotheses considered distances to formerly occupied roosts that related to predation risk (details in Table S1 of Supplementary Information). If an agent did not find a new roost prior to sunrise (< 5 min), it would have to enter its most recent roost. The final model of bat behaviour is illustrated in the state diagram (Fig. 2).

### Running of simulations

The process of construction and adjustments of the agent-based model was performed in the MATLAB (MathWorks, Inc) programming software (the Matlab code is in the Code file of Supplementary Information). Simulation of the behaviour of 100 agents using 3,456,000 steps (one season) was repeated six times to explore variations in simulated outputs. Simulation of one season using a desktop platform with Intel Xeon CPU W3670 3.20 GHz, 6 GB RAM lasted approximately 30 h.

### Statistical analyses

The output of the simulation was a list of daily occupied cavities with relevant group sizes as was found when bats’ roost switching activity was completed (*t* > 4:00). We paid close attention to roosts occupied by maximum groups, which comprise the highest number of individuals on a particular day, as those roosts should reflect the main patterns in roost site preference during the season.

Due to varying sampling techniques, providing either full (simulated data) or random (field data) evidence, and need for credible comparison of spatial patterns, we compared only locations of maximum groups in the simulations with roosts found in the field during the long-term study. We were confident that the field data set would also depict locations of the most preferred roosts of bats in the study area. With no need for concern about statistical concepts of randomness, uniformity and clustering, statistical comparison of the similarity of two such point patterns was made by an area-based nonparametric spatial point pattern test^58^ using a package ‘sppt’ 0.2.1^59^ of the R 3.6.3 environment for statistical computing^60^. To avoid possible overestimation of patterns’ similarity, we used the robust global *S*-values as a similarity index. The *S*-values were calculated only considering the number of hexagonal grid cells dividing the area that had roosts presently occurring within them, in either the field or in simulated data sets.

Simulated data such as group size, roost height and roost switching distance were compared with field collected values (n = 31 groups, 35 roosts, 21 switching events), though we were unable to compare other parameters obtained from simulations (reported only) due to a lack of evidence from the field. We calculated both unstandardized and standardized mean differences (Hedge’s *g*) between the simulated and observed natural data with 95% confidence intervals using the R packages ‘rcompanion’ 2.3.25^61^ and ‘effsize’ 0.8.0^62^, respectively. The magnitude of standardized effect size was assessed using the thresholds by Cohen^63^. Values of simulated and field sampling were compared also by a rank-based nonparametric Mann–Whitney *U* test using the core R package ‘stats’ 3.6.3^60^. Note that the switching distance in simulated data was based on maximum group roosts, whereas in field data it was based on harp-trapping of banded individuals. However, both methods considered switching between two consecutive days.

## Results

**Figure 3:**
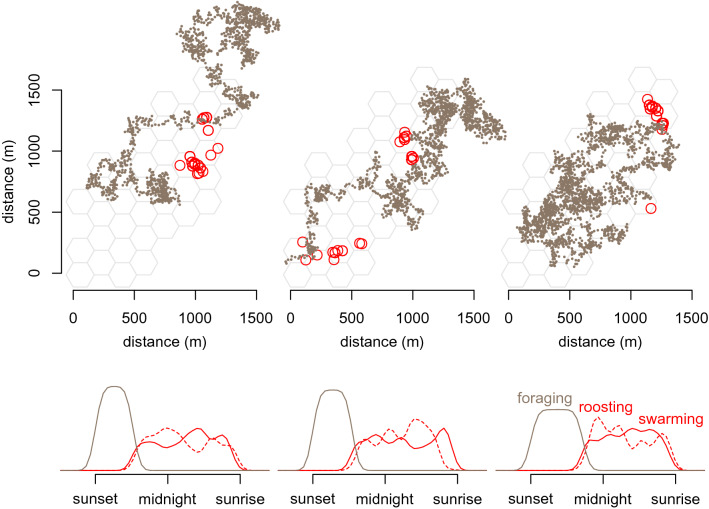
Simulated foraging trajectory (small points) and swarming around tree roosts (open circles) of three random agents (bats) during a single night in an area with properties as in Fig. 1. Density plots also denote time when bats were roosting in cavities.

**Figure 4:**
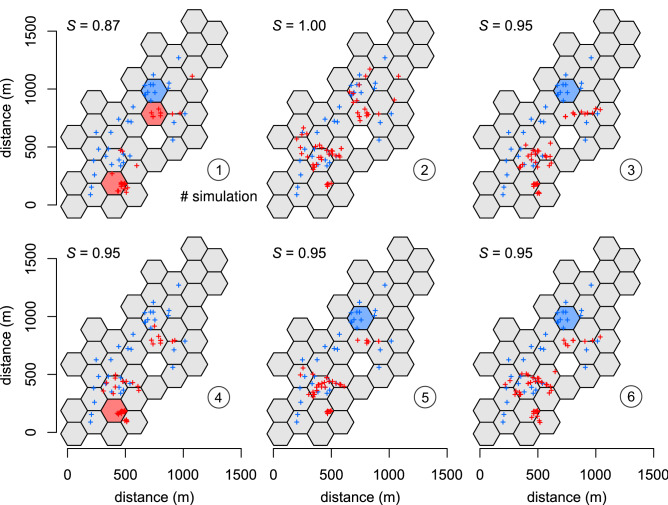
Spatial distribution of tree roosts (red crosses) of maximum groups, which could form from 100 possible agents during the six independent simulations of 120 days each. The number of agents and number of days were set to mimic natural population size of the Leisler’s bat in the area and the length of breeding season, respectively. Distribution of simulated roosts was compared with observed natural roosts (blue crosses) by an area-based nonparametric spatial point pattern test. Red cells of hexagonal grid refer to statistically significant increases (*P* < 0.05) of the number of simulated roosts, while blue cells refer to decreases (*S*, robust global index of similarity).

Individual movement and spatiotemporal distribution of swarming activity of simulated agents was highly variable (Fig. 3). However, simulated locations of roosts where groups were formed had very similar and often even identical spatial patterns to the locations of roosts observed in the field. For six independent simulations, the robust global *S*-values ranged from 0.87 to 1.00. Significant differences (*P* < 0.05) were seldom identified at the local level (Fig. 4). The majority of both simulated and field-observed roosts were located in the areas (hexagonal cells) of the highest density of available tree cavities (Fig. 1b).

**Figure 5:**
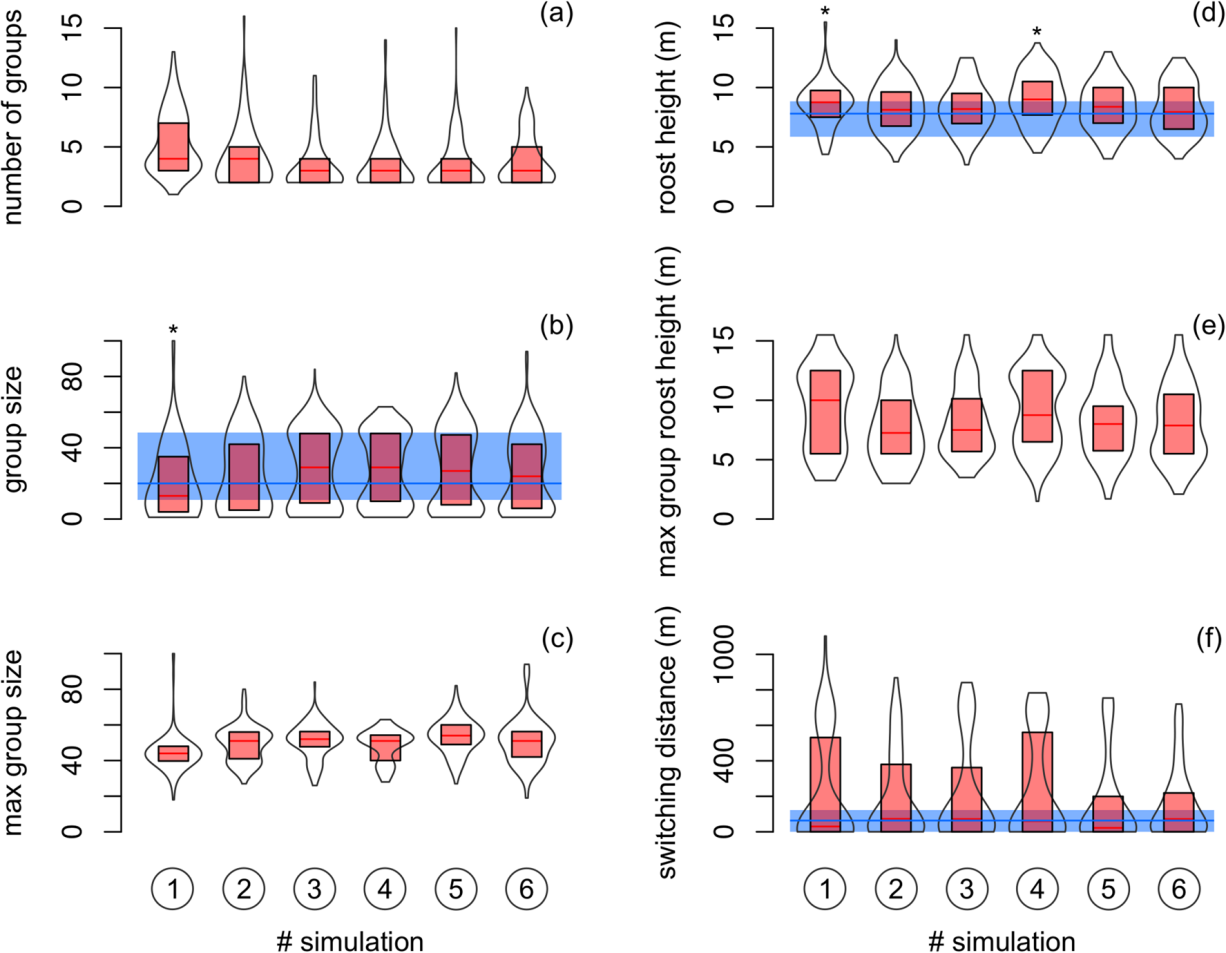
(**a**) Number of groups, (**b, c**) sizes of groups, (**d, e**) heights of roosts and **(f)** the distance between two roosts that were occupied by the maximum group in two consecutive days in the six independent simulations. Simulated data (violin plots show medians and 25–75% quartiles in box with rotated kernel density estimation) were compared with observed data from the natural population (horizontal median line with 25–75% quartiles) if allowed. Significant differences (*P* < 0.05) by the Mann–Whitney *U* test are denoted by asterisks.

The median number of simulated groups per day was 3 (total range of 1–16 of six independent simulations). The median size of such groups was 22 (1–100) individuals. The maximum group formed at the end of bats’ roost switching activity was about 51 (18–100) individuals. The median roost height was 8.5 (3.5–15.5) meters. Similarly, the roost height of maximum groups was about 8.3 (1.5–15.5) meters. Simulated switching distance had median 49 (range 0–1103) meters (Fig. 5). Simulated group size was in average 1.3–10 individuals smaller comparing to field data (total range of 95% CI was from – 17.5 to 5.8). The mean difference in simulated and observed roost height and switching distance was about 0.2–1.0 m (95% CI from – 0.4 to 1.7) and 58–133 m (95% CI from – 9 to 183), respectively. However, the magnitude of effect size in standardized mean differences between simulated and observed data was assessed as either small or even negligible (Table 1). Similarly to the effect size statistics, rank-based test indicated that simulated group size, roost height and switching distance differed from values observed in the field only seldom (Fig. 5).

**Table 1:** Mean difference between simulated and observed data in original unites and standardized measure of the effect size (Hedge’s *g*).

Simulation	Difference	95% CI		Hedge’s *g*	95% CI		Magnitude
**Group size**	[Individuals]						
1	– 10.0	– 17.5	– 2.5	– 0.49	– 0.85	– 0.13	Small
2	– 6.2	– 13.4	1.1	– 0.31	– 0.67	0.06	Small
3	– 1.3	– 8.3	5.8	– 0.06	– 0.43	0.31	Negligible
4	– 2.0	– 9.2	5.2	– 0.10	– 0.47	0.26	Negligible
5	– 1.8	– 8.8	5.2	– 0.08	– 0.44	0.28	Negligible
6	– 4.1	– 11.2	3.0	– 0.19	– 0.56	0.17	Negligible
**Roost height**	[m]						
1	0.7	0.0	1.3	0.31	– 0.07	0.69	Small
2	0.2	– 0.4	0.8	0.10	– 0.27	0.48	Negligible
3	0.3	– 0.3	0.9	0.13	– 0.25	0.51	Negligible
4	1.0	0.5	1.7	0.47	0.09	0.85	Small
5	0.4	– 0.2	1.0	0.17	– 0.21	0.55	Negligible
6	0.2	– 0.4	0.8	0.09	– 0.29	0.47	Negligible
**Switching distance**	[m]						
1	118	71	165	0.39	– 0.23	1.02	Small
2	88	29	146	0.37	– 0.26	0.99	Small
3	112	60	164	0.41	– 0.21	1.03	Small
4	133	83	183	0.47	– 0.15	1.09	Small
5	62	4	120	0.26	– 0.36	0.88	Small
6	58	– 9	126	0.30	– 0.32	0.92	Small

Both effect size statistics have shown lower and upper limits of 95% confidence intervals. The magnitude of effect size is assessed using the thresholds by Cohen^63^.

## Discussion

In the presented agent-based model, both the daily and seasonal patterns of swarming activity of agents resembled bats in their natural environment^35,36^. Incorporating selected parameters of the species and its environment, the SkyBat swarm algorithm^48,49^ competently replicated natural fission–fusion dynamics of tree-dwelling bat groups in predefined conditions. The low variation of means of computing data in six independent replications confirmed that the computational algorithm that processed biological input information produced consistent results. The most important outputs, which allowed us to evaluate the characteristics of agents’ fission–fusion behaviour, were the final number of simulated groups formed prior to the end of each simulation cycle (sunrise in the field) and the mean and maximum numbers of individuals in groups with respect to the local population size. This data did not differ significantly from the field data on Leisler’s bats collected during the long-term study in the Gavurky population (Table 1, Figs. 4 and 5).

Agents in the simulations behaved similarly to their biological source of inspiration, as groups selected cavity entrances more than 7.5 m above the ground, significantly higher than the average of available cavities in the study habitat^53^. For the study species, such roosting strategy suits reduced wing loading and lift-to-drag ratio^64^. Additionally, this strategy ensures required thermal conditions of roosts due to benefits arising from greater exposure of cavity to sunlight^4,24,25^ and reduces the risk of predation from the ground^10^. Furthermore, even switching distance between roosts was very similar to our field observations, and therefore we may consider simulation outputs as entirely plausible. Thus, our study confirmed that this algorithm is able to rule out a flexible non-centralized group decision while resolving multiple individual tasks during roost switching of tree-dwelling bats in a cluttered environment^5,36,38^. However, besides species-specific modeling of fission–fusion behaviour in bats, a more general implication of our findings could be for understanding of social dynamics that brings together different disciplines in a complex. We may thus study better interactions of individual group members as well the relationship between individual interactions and group level behaviours^65^.

Utilization of this modern computational approach in the study of dawn swarming behaviour of tree-dwelling bats is largely unknown. We are fully aware of very complex social behaviour and cognitive abilities of bats^22^, and our pioneering study can illuminate only a basic framework of the dynamic system of roost switching. We also did not account yet for the effect of additional variation in biological information at an individual level (e.g., age, relatedness, fitness, hormonal secretion) and how that data would improve the model, but our case study is vital for understanding the roost switching of bat groups in a rapidly changing environment. Together with changed characteristics of roosts, one of the proximate factors shaping fission–fusion dynamics are indeed ambient conditions as females are more likely to switch roosts at high temperatures while less when it rained^23,26^. Further, for example, by modelling specific conditions of the species’ forest habitat, one can also predict the response of the local population to logging practice or altered food supply. We therefore suggest that the SkyBat can be employed as a tool for biological conservation.

Results of this interdisciplinary study also suggest possible applications of the algorithm not only in bat ecology but also in the fields of computer science and artificial intelligence. In all fairness, besides other bio-inspired, swarm-based computational algorithms characterized by convergent social phenomena in different animals, a bat algorithm with derivates has already been described^66^. Although this computational algorithm has the unique ability to solve complex problems^67–70^, its formal description was solely inspired by echolocation properties during group foraging. The grouping mechanism in foraging individuals is completely different from group roosting, while group foraging is associated with even less investigated reasons for sociality in bats^71,72^. The most important features of a swarming algorithm applied in the SkyBat model are its ability to find a solution in a limited time and its independence from the environment and specific relationships among individuals. A derivative of the SkyBat algorithm was successfully tested as a searching algorithm in a field of informatics^49^. Furthermore, a highly potential candidate for utilization of the SkyBat is swarm robotics, a relatively new bio-inspired approach that aims to coordinate a large number of autonomous robotic agents in searching for specific targets of interest and moving groups from one location to another without a leader^73^. Thus, the coordination among robotic agents is achieved in a self-organised manner where their collective behaviour is a result of local interactions among them and between them and their environment. This is directly comparable to fission–fusion dynamics in social groups of tree-dwelling bats.

## Supplementary information

Supplementary Code

Supplementary Information.

## Data Availability

The data sets generated and analysed in the study are available from the corresponding author upon request.
