# Supplementary material for: An agent-based algorithm resembles behaviour of tree-dwelling bats under fission–fusion dynamics
Source: Sci Rep. 2020 Oct 8;10:16793. doi: 10.1038/s41598-020-72999-0 (PMC7545098; doi:10.1038/s41598-020-72999-0)
Supplement: Supplementary file 2 — Supplementary Information. [file 41598_2020_72999_MOESM2_ESM.doc]

**Supplementary Information**

**An agent-based algorithm resembles behaviour of tree-dwelling bats under fission-fusion dynamics**

Ján Zelenka^1^, Tomáš Kasanický^1^, Ivana Budinská^1^ & Peter Kaňuch^2*^

^1^Institute of Informatics, Slovak Academy of Sciences, 845 07 Bratislava, Slovakia.

^2^Institute of Forest Ecology, Slovak Academy of Sciences, 960 53 Zvolen, Slovakia.

*e-mail: kanuch@netopiere.sk

**
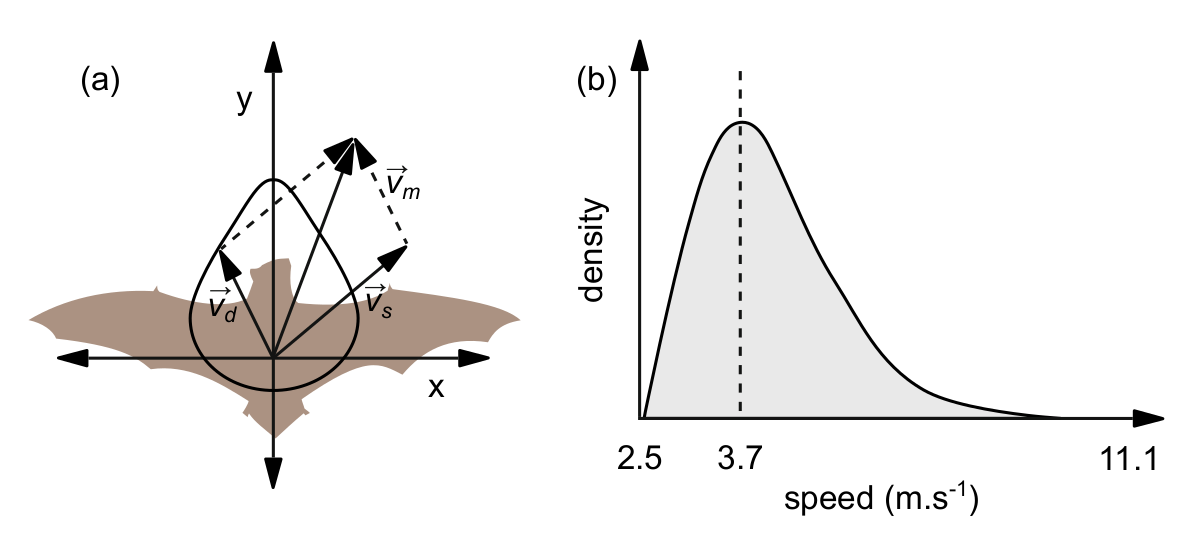
**

**Figure S1.** Movement of bat agents. Predetermined directional **(a)** and speed distribution **(b)** responsible for highly correlated random walk of bats.


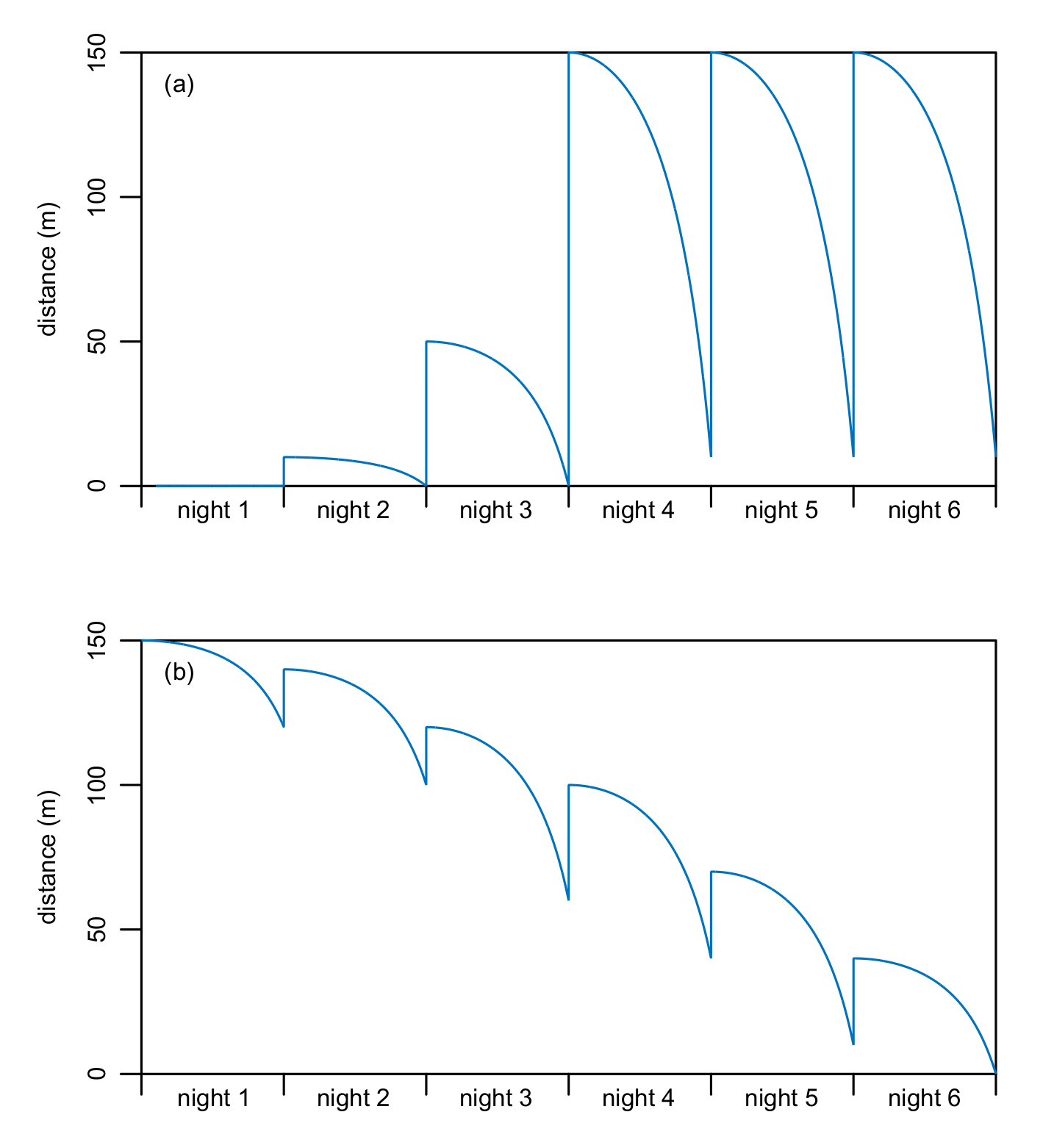


**Figure S2.** The *PD* radius function for roost penalization considering the risk of predation, for actual **(a)** and previous roost **(b)**.

**Table S1.** Inspection of an unattracted cavity by a bat is activated if following hypotheses are fulfilled (×). Higher value is the higher probability for inspection. H1 – cavity is located in the field of perception of the bat; H2 – cavity was occupied by the bat in the past (or it was only visited during swarming); H3 – no risk of predation in the actual roost (Fig. S2a); H4 – no risk of predation due to previous roosting (Fig. S2b); H5 – cavity is no more than 800 m from the previous roost (this distance is set according to real limits of the study area)

| H1 | H2 | H3 | H4 | H5 | value |
| --- | --- | --- | --- | --- | --- |
| × | × | × | × | × | 15 |
| × | (×) | × | × | × | 14 |
| × |  | × | × | × | 13 |
| × | × |  | × | × | 12 |
| × | (×) |  | × | × | 11 |
| × |  |  | × | × | 10 |
| × | × | × | × |  | 12 |
| × | (×) | × | × |  | 11 |
| × |  | × | × |  | 10 |
| × | × |  | × |  | 9 |
| × | (×) |  | × |  | 8 |
| × |  |  | × |  | 7 |
| × | × | × |  | × | 12 |
| × | (×) | × |  | × | 11 |
| × |  | × |  | × | 10 |
| × | × |  |  | × | 9 |
| × | (×) |  |  | × | 8 |
| × |  |  |  | × | 7 |
| × | × | × |  |  | 9 |
| × | (×) | × |  |  | 8 |
| × |  | × |  |  | 7 |
| × | × |  |  |  | 6 |
| × | (×) |  |  |  | 5 |
| × |  |  |  |  | 4 |
